# 

**Published:** 1859-09

**Authors:** 


### DENTAL REGISTER

### OF THE WEST.

EDITED BY

J. TAFT AND GEO. WATT.

September, 1859.

MONTHLY.--THREE DOLLARS PER ANNUM, IN ADVANCE.

CINCINNATI:

J. T. TOLAND, PUBLISHER AND PROPRIETOR.

1859.

